# Phthalates Induce Neurotoxicity Affecting Locomotor and Thermotactic Behaviors and AFD Neurons through Oxidative Stress in *Caenorhabditis elegans*

**DOI:** 10.1371/journal.pone.0082657

**Published:** 2013-12-12

**Authors:** I-Ling Tseng, Ying-Fei Yang, Chan-Wei Yu, Wen-Hsuan Li, Vivian Hsiu-Chuan Liao

**Affiliations:** Department of Bioenvironmental Systems Engineering, National Taiwan University, Taipei, Taiwan; McGill University, Canada

## Abstract

**Background:**

Phthalate esters are ubiquitous environmental contaminants and numerous organisms are thus exposed to various levels of phthalates in their natural habitat. Considering the critical, but limited, research on human neurobehavioral outcomes in association with phthalates exposure, we used the nematode *Caenorhabditis elegans* as an *in vivo* model to evaluate phthalates-induced neurotoxicity and the possible associated mechanisms.

**Principal Findings:**

Exposure to phthalates (DEHP, DBP, and DIBP) at the examined concentrations induced behavioral defects, including changes in body bending, head thrashing, reversal frequency, and thermotaxis in *C. elegans*. Moreover, phthalates (DEHP, DBP, and DIBP) exposure caused toxicity, affecting the relative sizes of cell body fluorescent puncta, and relative intensities of cell bodies in AFD neurons. The mRNA levels of the majority of the genes (TTX-1, TAX-2, TAX-4, and CEH-14) that are required for the differentiation and function of AFD neurons were decreased upon DEHP exposure. Furthermore, phthalates (DEHP, DBP, and DIBP) exposure at the examined concentrations produced elevated intracellular reactive oxygen species (ROS) in *C. elegans*. Finally, pretreatment with the antioxidant ascorbic acid significantly lowered the intracellular ROS level, ameliorated the locomotor and thermotactic behavior defects, and protected the damage of AFD neurons by DEHP exposure.

**Conclusions:**

Our study suggests that oxidative stress plays a critical role in the phthalate esters-induced neurotoxic effects in *C. elegans*.

## Introduction

Endocrine-disrupting chemicals (EDC) are exogenous compounds that have the potential to alter hormonal and homeostatic systems, thereby affecting health and reproduction in animals and humans [1]–[4]. Several toxicity studies have shown that exposure to EDC lowers fertility, through premature ovarian failure, reproductive tract anomalies, and poor semen quality [5]–[7]. Prenatal and postnatal exposure to bisphenol A produced abnormalities in learning and memory in mice [8]. EDC-like phthalates are ubiquitous in the environment and in human tissues [9]. The term “phthalate” is used to refer to a dialkyl ester of ortho-phthalic acid in which the alkyl portion is a hydrocarbon chain with one or more carbon atoms. Because phthalates are widely used in daily life, human exposure is nearly universal. The most commonly used phthalates are bis(2-ethylhexyl) phthalate (DEHP), dibutyl phthalate (DBP), and diisobutyl phthalate (DIBP), which are used as plasticizers, solvents, and additives in numerous consumer products, such as vinyl flooring, food containers, cosmetics, pharmaceuticals, and children's toys [10], [11]. Phthalates were postulated to produce endocrine-disrupting effects in rodents; animal research strongly implicated high-dose exposure to certain phthalates (DEHP, DBP, and BBP) altered developmental and reproductive functions in rats [12]. The observed adverse effects in rodent models raise concerns about whether phthalate exposure poses a potential health risk to humans.

There is limited, but growing, evidence linking exposure to certain phthalates with neurobehavioral outcomes. Studies of neurobehavioral outcomes for humans, following phthalate exposure, are limited. In animals, a number of studies have reported that phthalate exposure was associated with altered neurobehaviors, including impaired self-righting ability [13], [14], impaired spatial learning and reference memory [13], [15], increased hyperactivity [16], and decreased grooming behavior [17]. Moreover, several studies have reported that phthalate exposure is associated with deficits in social functions [18], reduced intelligence [19], attention deficit hyperactivity disorder (ADHD) at school age [20], and autism spectrum disorders (ASDs) [21]. Recent studies have shown that prenatal phthalate exposure is associated with alterations in childhood behavior and executive functioning [22], [23].

Given the vital but limited research on human neurobehavioral outcomes in association with phthalate exposure and the potentially substantial public health impact of the pervasiveness of phthalates in our environment, our objective was to use the nematode *Caenorhabditis elegans* as an *in vivo* model to evaluate phthalate-induced neurotoxicity. *C. elegans* has been established as a model for studying neurotoxicity because it contains 302 neurons and its neuronal lineage is fully described [24], [25]. Neurotransmitter systems, including serotonergic, cholinergic, glutamatergic, and γ-aminobutyric acid (GABA)-ergic synapses, and their genetic networks, are phylogenetically conserved from nematodes to vertebrates, which allow findings from *C. elegans* to be extrapolated and further confirmed in vertebrate systems [25].

Phthalates might induce neurotoxicity, but little is known about the mechanisms by which this occurs. In this study, we selected 3 commonly used phthalates (DEHP, DBP, and DIBP) to investigate phthalate-induced neurotoxic effects on locomotor and thermotactic behaviors, and AFD thermosensory neurons in *C. elegans*. In addition, we investigated the possible mechanisms involved in these effects.

## Results

### Effects of phthalates exposure on locomotor behaviors in *C. elegans*

Phthalate esters may induce neurotoxicity. We examined neurotoxicity induced by phthalates DEHP, DBP, and DIBP, and the possible associated mechanisms in *C. elegans*. First, we examined the effects of DEHP, DBP, and DIBP exposure on locomotor behaviors. Locomotor behavioral assays, including the numbers of body bends, head thrashes, and the reversal frequency are well established protocols for studying neuronal circuits that control behaviors [26]. To test whether DEHP, DBP, and DIBP have toxic effects on locomotor behaviors in *C. elegans*, body bending, head thrashing, and reversal frequency were examined.

**Figure 1:**
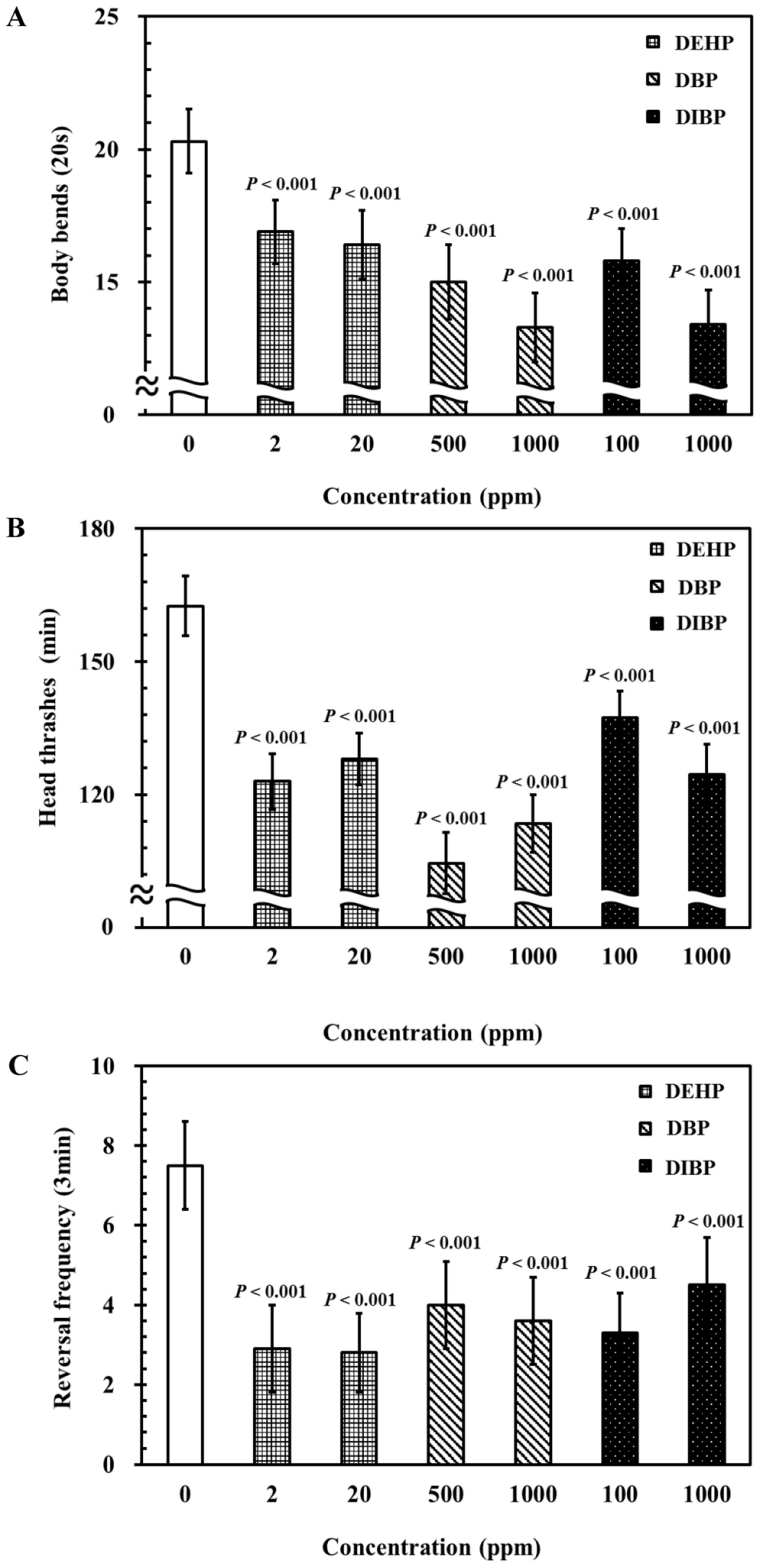
Effects of phthalates exposure on locomotor behaviors in *C. elegans*. Synchronized wild-type L1 larvae were incubated in liquid S-basal containing *E. coli* OP50 bacteria, at 10^9^ cells/mL or 0.1% ethanol as the solvent control, for 40 h, at 20°C. Subsequently, L4-stage nematodes were incubated in K-medium, with or without DEHP (2 and 20 ppm), DBP (500 and 1000 ppm), and DIBP (100 and 1000 ppm) for 24 h at 20°C. Adult worms were selected for analysis of the rate of locomotion. (A) The number of body bends in 20 s, (B) the number of head thrashes in 1 min, and (C) the reversal frequency in 3 min. Approximately 30 worms from each treatment, at each time point, were randomly selected for scoring. The tests were performed a minimum of 3 times. The results were presented as the mean ± standard errors of mean (SEM). Differences compared to the control (0 ppm, 0.1% ethanol) were considered significant at *P*<0.05 by one-way ANOVA and the LSD post-hoc test.

For body bend analysis, L4-stage wild-type worms were exposed to various concentrations of commonly used phthalates (DEHP (2 and 20 ppm), DBP (500 and 1000 ppm), and DIBP (100 and 1000 ppm)) for 24 h at 20°C. The results showed that all of the examined concentrations of DEHP, DBP, and DIBP caused significant reductions in the number of body bends compared with those of non-exposed control worms (Figure 1A).

Similarly, a substantial decrease in head thrashing occurred in worms exposed to DEHP (2 and 20 ppm), DBP (500 and 1000 ppm), and DIBP (1000 ppm), compared with non-exposed control worms (Figure 1B). Moreover, while L4-larval stage nematodes were exposed to DEHP, DBP, and DIBP for 24 h, a significant decrease in reversal frequency was observed in worms in all examined concentrations of DEHP, DBP, and DIBP, compared with the non-exposed control worms (Figure 1C). When L4-larval stage nematodes were exposed to DEHP at a concentration of 2 ppm, significant (*P*<0.001) body bend, head thrash, and reversal frequency defects were observed, whereas the adult nematodes required higher concentrations of DBP (500 ppm) and DIBP (100 ppm) exposure to exhibit similar defects. The results indicate that the phthalates DEHP, DBP, and DIBP can cause locomotor behavior defects in *C. elegans*, and that DEHP is the most toxic of these phthalates.

### Effects of phthalates exposure on thermotactic behaviors in *C. elegans*

**Figure 2:**
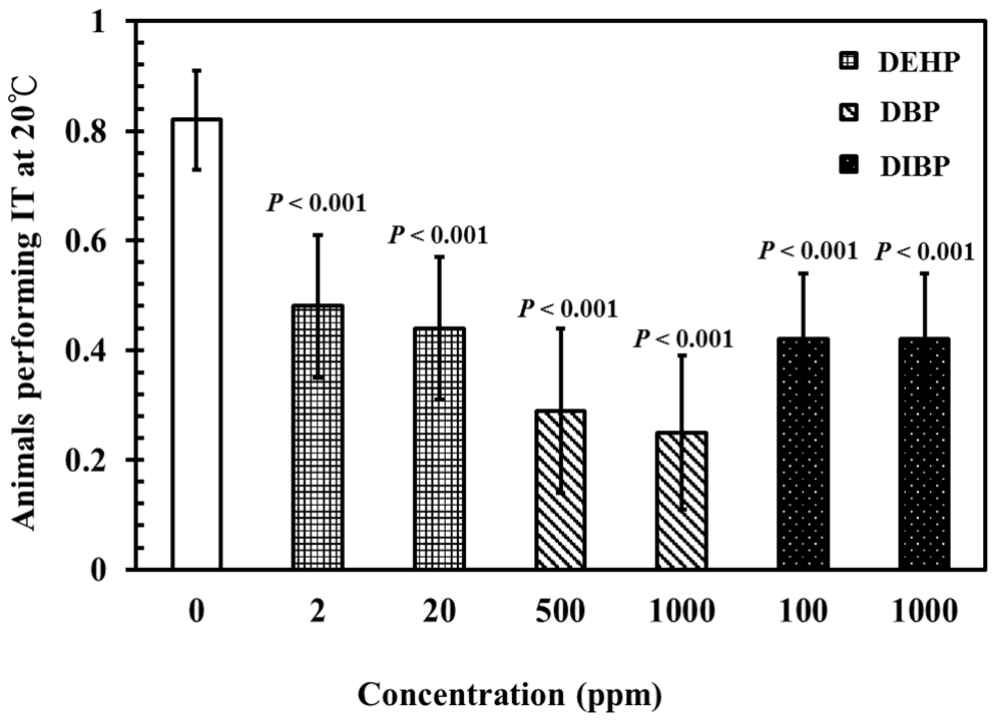
Effects of phthalates exposure on thermotaxis in *C. elegans*. Synchronized wild-type L1 larvae were incubated in liquid S-basal containing *E. coli* OP50 bacteria, at 10^9^ cells/mL or 0.1% ethanol as the solvent control, for 40 h, at 20°C. Subsequently, L4-stage nematodes were incubated in K-medium, with or without DEHP (2 and 20 ppm), DBP (500 and 1000 ppm), and DIBP (100 and 1000 ppm) for 24 h at 20°C. Adult worms were selected for thermotactic analysis. Thermotaxis was evaluated by the percentage of worms performing isothermal tracking (IT) behavior at the cultured temperature (20°C). A trace is considered as isothermal if more than half of the trace length left on the agar surface by a single nematode is circular or presents an arc near the isotherm of the growth temperature. Each datum represents a minimum of 30 independent assays. The results were presented as the mean ± standard errors of mean (SEM). Differences compared to the control (0 ppm, 0.1% ethanol) were considered significant at *P*<0.05 by one-way ANOVA and the LSD post-hoc test.

Previous studies have shown that the interneurons of the thermotactic network form widespread and redundant anatomical connections with the interneurons for reversals [27], [28]. Therefore, we investigated thermotactic behaviors in *C. elegans* exposed to phthalates. L4-stage wild-type worms were treated with various concentrations of DEHP (2 and 20 ppm), DBP (500 and 1000 ppm), and DIBP (100 and 1000 ppm) for 24 h at 20°C. Subsequently, thermotactic behavior was evaluated according to the percentage of worms performing isothermal tracking (IT) behavior at cultivation temperature (20°C). As shown in Figure 2, exposure to DEHP (20 ppm), DBP (500 and 1000 ppm), and DIBP (100 and 1000 ppm) caused a significant decrease in the percentage of IT behaviors in *C. elegans* at 20°C, compared with the controls. This suggests that exposure to phthalate-related compounds causes severe defects in nematodes at the cultivation temperature (20°C).

### Effects of phthalates exposure on AFD neurons in *C. elegans*

**Figure 3:**
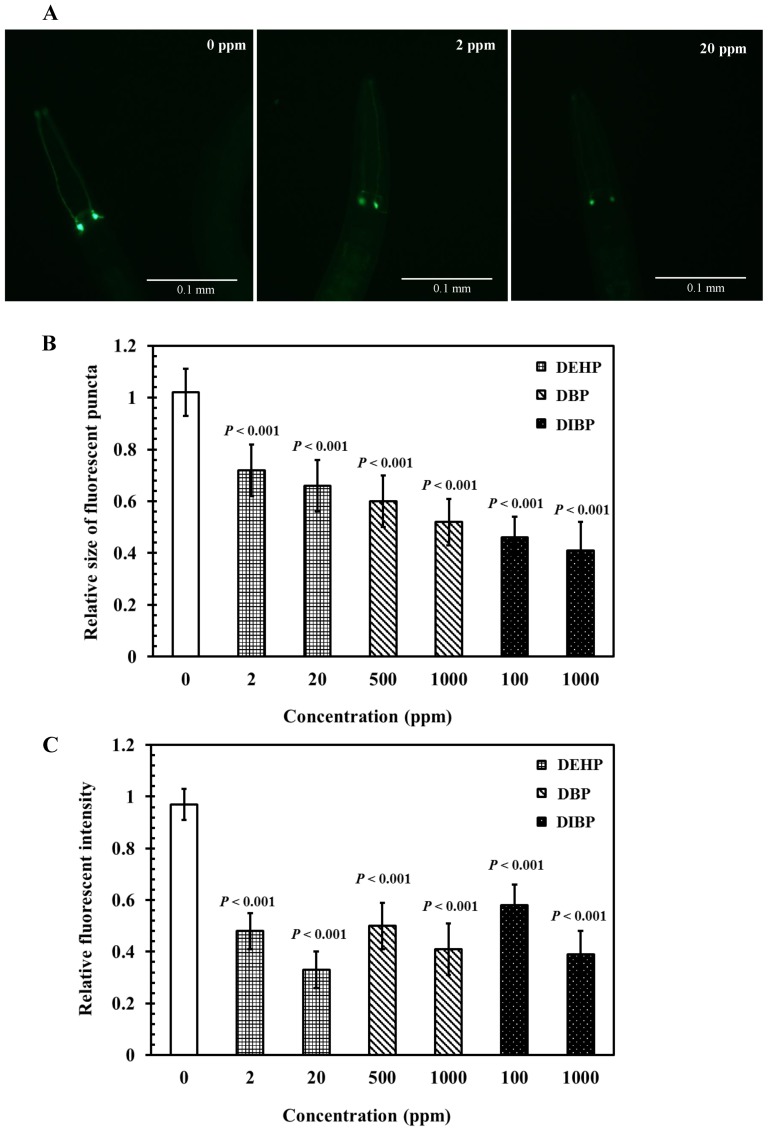
Effects of phthalates exposure on AFD thermosensory neurons in *C. elegans*. Synchronized DA1267 L1 larvae were incubated in liquid S-basal containing *E. coli* OP50 bacteria, at 10^9^ cells/mL or 0.1% ethanol as the solvent control, for 40 h, at 20°C. Subsequently, L4-stage nematodes were incubated in K-medium, with or without DEHP (2 and 20 ppm), DBP (500 and 1000 ppm), and DIBP (100 and 1000 ppm), for 24 h at 20°C. (A) Representative images of morphological patterns of AFD sensory neurons labeled with P*gcy-8*::GFP, after DEHP exposure. (B) Relative sizes of fluorescent puncta for cell bodies of AFD sensory neurons. (C) Relative fluorescence intensities in cell bodies of AFD sensory neurons. Relative sizes of fluorescent puncta and relative fluorescence intensities were calculated by normalizing to that of the control. Approximately 30 worms from each treatment, at each time point, were randomly selected for analysis. The tests were performed a minimum of 3 times. The results were presented as the mean ± standard errors of mean (SEM). Differences compared to the control (0 ppm, 0.1% ethanol) were considered significant at *P*<0.05 by one-way ANOVA and the LSD post-hoc test.

Thermosensation-associated learning and memory rely on AFD neurons [29]. In *C. elegans*, P*gcy-8*::GFP is a specific fluorescent marker that labels the AFD sensory neurons [30]. We examined the relative fluorescence intensities and relative sizes of cell body fluorescent puncta in cell bodies in AFD sensory neurons of worms (P*gcy-8*::GFP). Transgenic L4-stage worms (P*gcy-8*::GFP) were treated with various concentrations of DEHP (2 and 20 ppm), DBP (500 and 1000 ppm), and DIBP (100 and 1000 ppm) for 24 h at 20°C. Subsequently, the size of fluorescent puncta, and the relative fluorescence intensities in cell bodies in AFD sensory neurons were evaluated. Figure 3A shows the representative images of morphological patterns of AFD sensory neurons labeled with P*gcy-8*::GFP, after DEHP exposure. Exposure to DEHP (2 and 20 ppm), DBP (500 and 1000 ppm), and DIBP (100 and 1000 ppm) caused a significant reduction of relative sizes of cell body fluorescent puncta in AFD neurons in *C. elegans*, compared with controls (*P*<0.001) (Figure 3B). Similarly, exposure to DEHP (2 and 20 ppm), DBP (500 and 1000 ppm), and DIBP (100 and 1000 ppm) caused a substantial decrease in fluorescence intensities in cell bodies in AFD neurons, compared with controls (*P*<0.001) (Figure 3C). The results suggest that the size of fluorescent puncta and cell bodies in AFD neurons can be severely altered by exposure to phthalate-related compounds.

### Phthalates decrease mRNA levels of TTX-1, TAX-2, TAX-4, and CEH-14

We further examined the expression of genes (TTX-1, TAX-2, TAX-4, and CEH-14) that are required for the differentiation and function of AFD neurons, which might be affected by phthalate exposure. DEHP at a concentration of 2 ppm was selected because it was the lowest observed adverse effect concentration (LOAEC) to cause a severely altered size of fluorescent puncta and cell bodies in AFD neurons (Figure 3). TTX-1 is a transcription factor that mediates the expression of *gcy-8* [30]. The cyclic nucleotide-gated channels α-subunit, TAX-4, and β-subunit, TAX-2, are theorized to function directly in sensory transduction and to mediate several sensory behaviors [31], [32]. The LIM homeobox gene CEH-14 is required for the correct functioning of AFD neurons [33]. We examined the changes of mRNA levels of TTX-1, TAX-2, TAX-4, and CEH-14 in DEHP-exposed and control worms, by using real-time RT–PCR assays.

**Figure 4:**
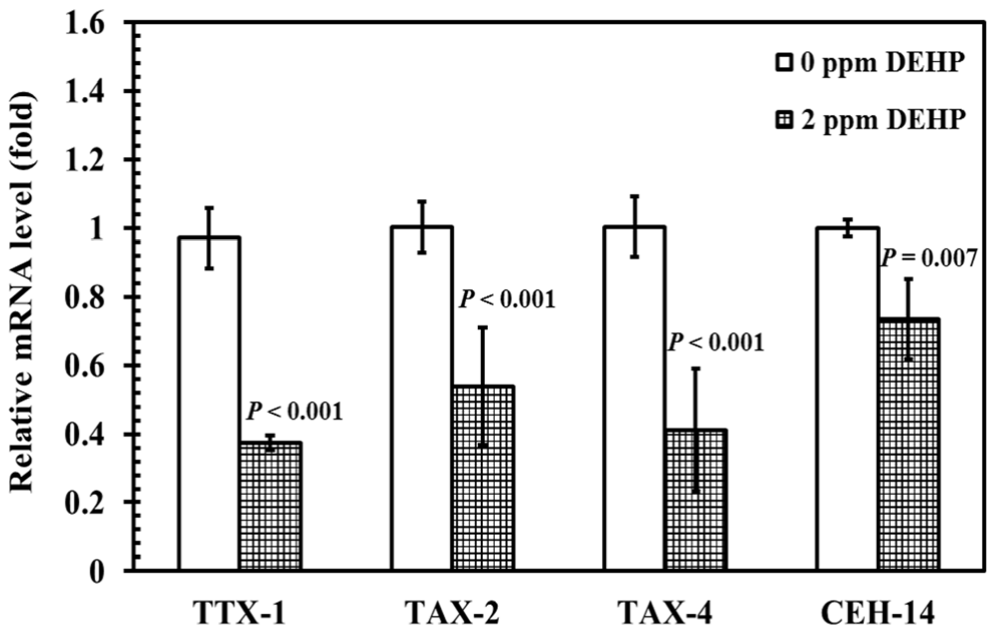
Effects of phthalates on the expression of TTX-1, TAX-2, TAX-4, and CEH-14 in *C. elegans*. Total RNA of wild-type worms under the condition of thermotaxis assay was extracted. The mRNA levels of TTX-1, TAX-2, TAX-4, and CEH-14 were determined using quantitative real-time RT-PCR. The mRNA levels were normalized to the expression of Y45F10D.4 [56]. The fold change was normalized to that observed in untreated solvent control samples. The test was performed 3 times. The results were presented as the mean ± standard errors of mean (SEM). Differences compared to the control (0 ppm, 0.1% ethanol) were considered significant at *P*<0.05 by one-way ANOVA and the LSD post-hoc test.

The results showed that when worms were exposed to 2 ppm of DEHP, the mRNA levels of TTX-1 (40%, *P*<0.001), TAX-2 (50%, *P*<0.001), TAX-4 (50%, *P*<0.001), and CEH-14 (70%, *P* = 0.007) were significantly decreased, compared to those in the control group (Figure 4). The results suggest that DEHP exposure influences the expression of many genes that are required for the differentiation and function of AFD neurons in *C. elegans*.

### Phthalates increase the intracellular reactive oxygen species level in *C. elegans*

We explored a mechanism that might explain the manner in which phthalates caused the neurotoxicity observed in Figures 1–4. Recent studies suggested that increased oxidative stress is related to severely impaired learning behavior and modestly reduced motor activity [34], [35]. Therefore, we hypothesized that reactive oxygen species (ROS) mediated oxidative damage is a critical factor that might explain the manner in which phthalates caused neurotoxicity in *C. elegans*. First, we examined the influence of DEHP, DBP, and DIBP exposure on intracellular ROS level in *C. elegans*.

**Figure 5:**
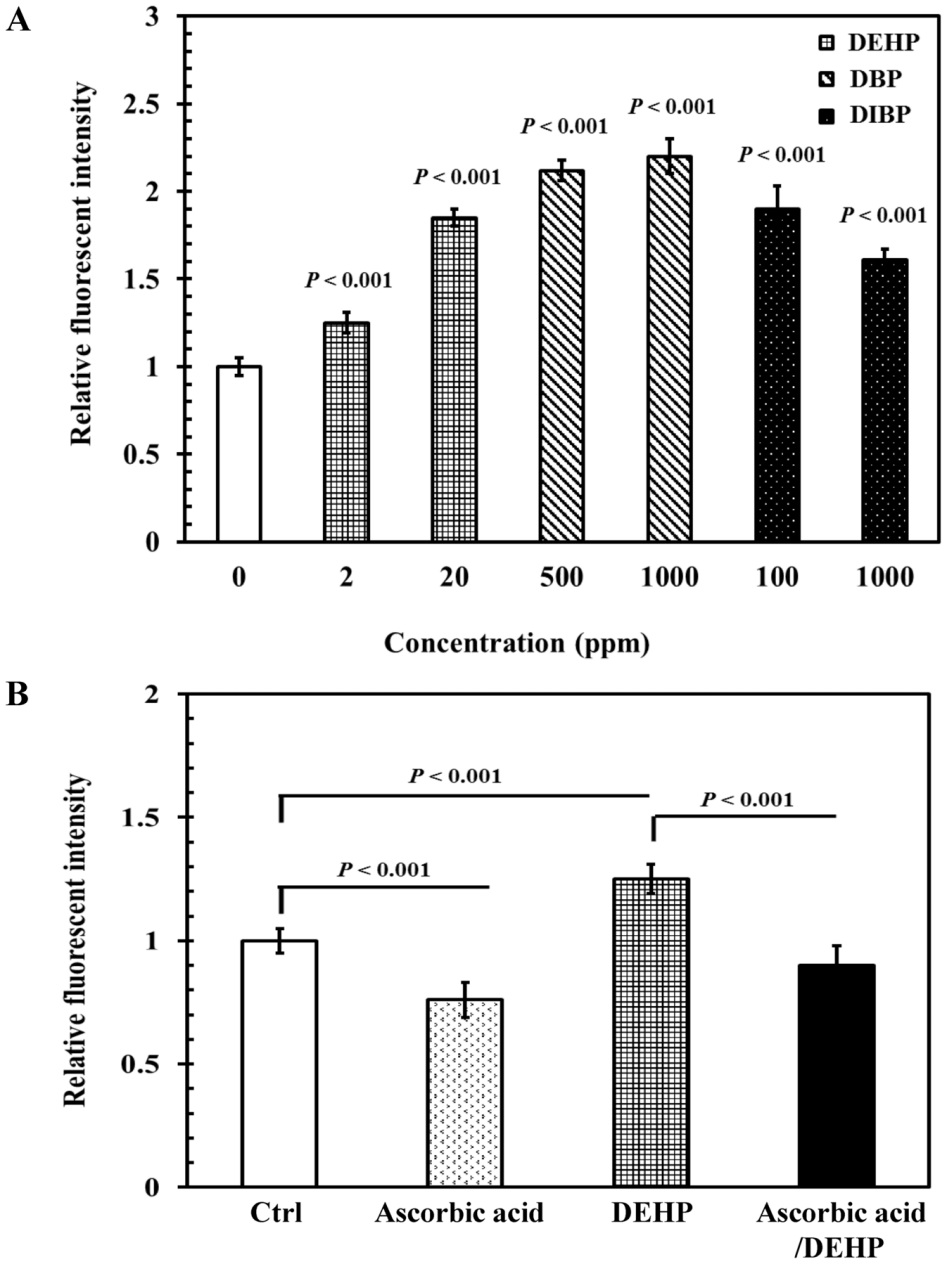
Effects of phthalates exposure on intracellular reactive oxygen species production in *C. elegans*. Synchronized wild-type L1 larvae were incubated in liquid S-basal containing *E. coli* OP50 bacteria, at 10^9^ cells/mL in the (A) absence or (B) presence of 250 µM ascorbic acid, or 0.1% ethanol as the solvent control, for 40 h, at 20°C. L4-stage worms were subsequently incubated in K-medium with or without phthalates for 24 h at 20°C. Subsequently, intracellular ROS for adult worms were measured using 2′, 7′-dichlorodihydrofluoroscein diacetate. One hundred worms from each exposure condition were used to analyze the intracellular ROS levels. The results are expressed as relative fluorescence units (RFU) of fluorescence relative to 100 worms. The tests were performed a minimum of 3 times. The results were presented as the mean ± standard errors of mean (SEM). Differences compared to the control (0 ppm, 0.1% ethanol) (A) or differences between the populations (B) were considered significant at *P*<0.05 by one-way ANOVA and the LSD post-hoc test.

To assay the effects of DEHP, DBP, and DIBP exposure on the intracellular ROS level, wild-type worms were raised from L1 larvae, as described in the locomotor and thermotactic behaviors assays, and L4 larval-stage worms were exposed to DEHP, DBP, and DIBP for 24 h. Subsequently, intracellular ROS in adult worms was measured using the CM-H_2_DCF-DA method. Non-fluorescent DCF-DA is a cell-permeable dye that can be readily converted to DCF, due to the interaction with intracellular peroxide (H_2_O_2_). As shown in Figure 5A, when worms were exposed to all examined concentrations of DEHP, DBP, and DIBP, the intracellular ROS level was significantly increased, compared with that in the control.

We further evaluated whether antioxidant treatment is able to inhibit phthalates-enhanced ROS production. Given DEHP at a concentration of 2 ppm was the LOAEC to cause the neurotoxicity observed in Figures 1–4, 2 ppm DEHP was selected for subsequent experiments. The results showed that antioxidant ascorbic acid (250 µM) [36] pretreatment significantly decreased the DEHP-evaluated ROS level compared with that for only DEHP treatment (2 ppm) (*P*<0.001) (Figure 5B). This implies that exposure to DEHP, DBP, and DIBP induced a significant increase of intracellular ROS production, which may damage the nervous systems in *C. elegans*.

### Antioxidant pretreatment suppresses locomotor and thermotactic behaviors induced by DEHP exposure in *C. elegans*

To elucidate the relationship between ROS production and phthalates-induced locomotor and thermotactic behavior defects in *C. elegans*, we treated wild-type nematodes with the antioxidant ascorbic acid (250 µM) [36] to ameliorate phthalates-induced locomotor and thermotactic behavior defects.

**Figure 6:**
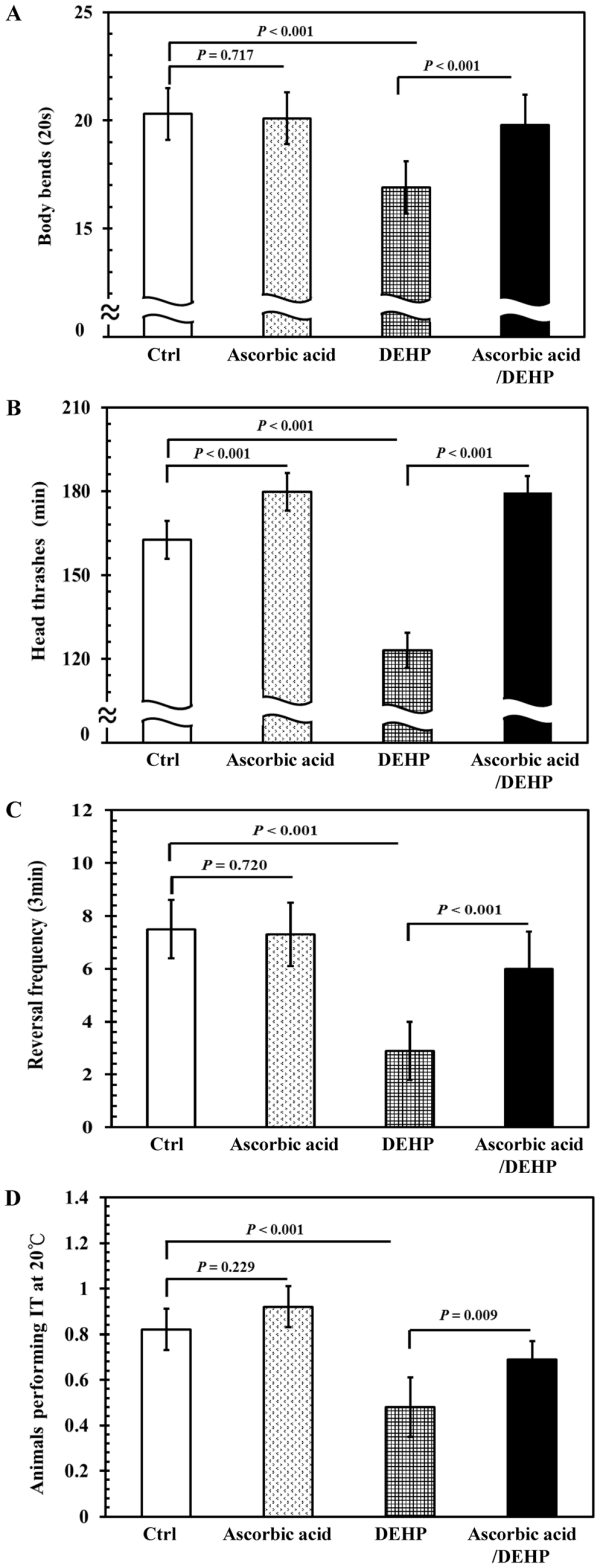
Effects of antioxidant pretreatment on locomotor and thermotactic behaviors in DEHP-exposed nematodes. Synchronized L1 wild-type larvae were incubated with 250 µM of ascorbic acid or 0.1% ethanol as the solvent control for 40 h at 20°C. Subsequently, ascorbic acid-pretreated and control worms were divided into two aliquots and treated with or without 2 ppm of DEHP for 24 h at 20°C. (A) The number of body bends in 20 s, (B) the number of head thrashes in 1 min, (C) the reversal frequency in 3 min, and (D) thermotaxis using the percentage of worms performing isothermal tracking (IT) behavior at 20°C. Approximately 30 worms from each treatment at each time point were randomly selected for scoring. The tests were performed a minimum of 3 times. The results were presented as the mean ± standard errors of mean (SEM). Differences between the populations were considered significant at *P*<0.05 by one-way ANOVA and the LSD post-hoc test. “Ctrl”, worms grown on a normal diet; “Ascorbic acid”, worms grown with ascorbic acid supplementation; “DEHP”, worms grown on a normal diet followed by DEHP exposure; “Ascorbic acid/DEHP”, worms with ascorbic acid pretreatment and followed by DEHP exposure.

Synchronized wild-type L1 larvae were raised in the presence or absence of ascorbic acid (250 µM) for 40 h, at 20°C. A subsequent DEHP exposure at a concentration of 2 ppm, was performed for 24 h. As shown in Figure 6A, pretreatment of nematodes during the L1 larval stage, using ascorbic acid for 40 h, significantly increased in body bends when compared with those without ascorbic acid pretreatment (*P*<0.001), suggesting that antioxidant ascorbic acid can counteract the toxicity induced by DEHP. Similarly, nematodes with ascorbic acid pretreatment exhibited significant protection against DEHP-induced toxicity on head thrash, reversal frequency, and thermotactic behaviors (Figures 6B, 6C, and 6D). Taken together, a pretreatment using antioxidant ascorbic acid can protect the locomotor and thermotactic behaviors of *C. elegans* by reducing the accumulation of intracellular ROS levels induced by DEHP, which may damage the nervous systems. This suggests a link between DEHP-induced oxidative stress and locomotor and thermotactic behavior defects in *C. elegans*.

### Antioxidant pretreatment protects AFD sensory neurons from DEHP-induced neuronal damage in *C. elegans*

**Figure 7:**
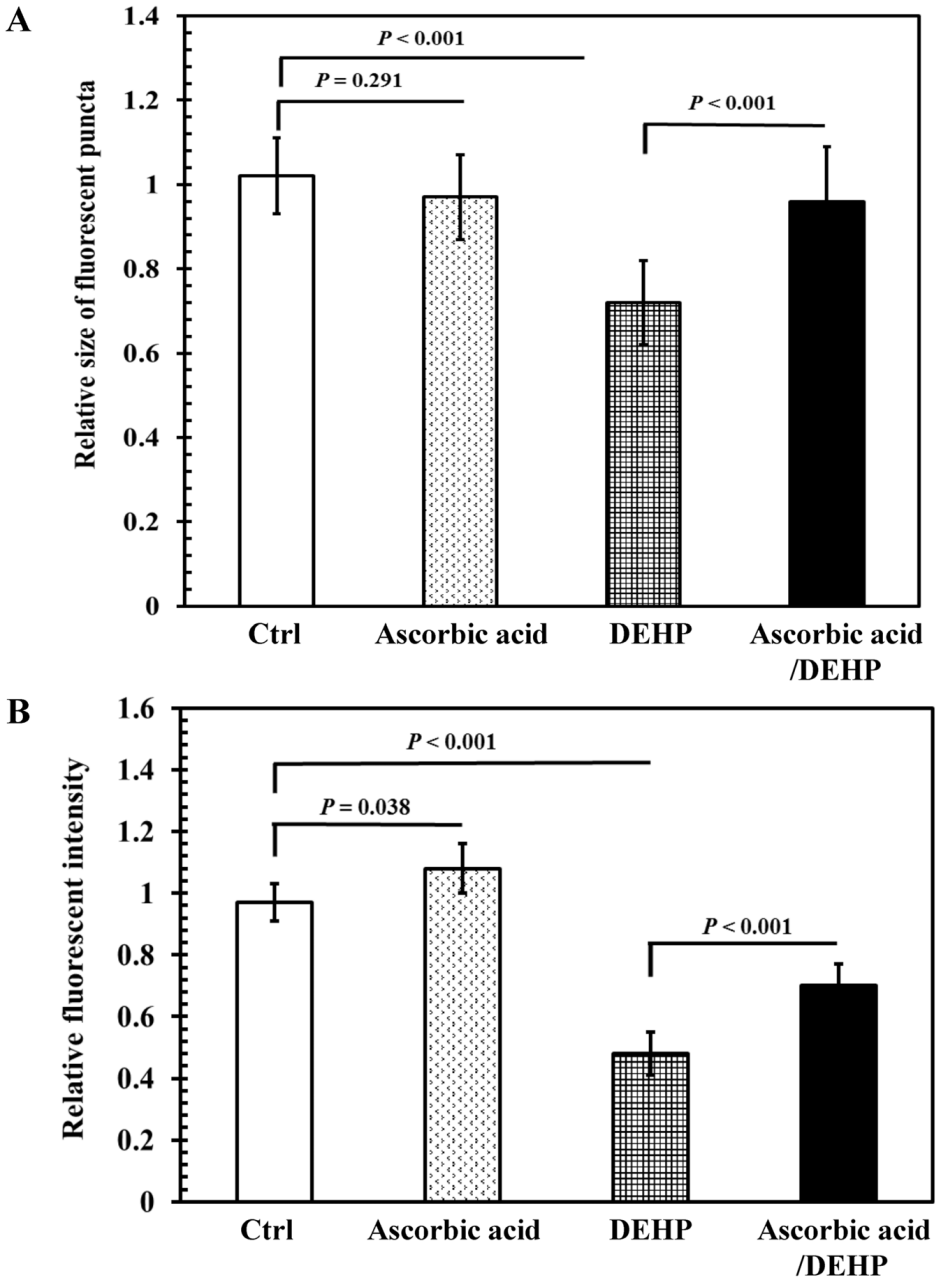
Effects of antioxidant pretreatment on AFD thermosensory neurons in DEHP-exposed nematodes. Synchronized DA1267 L1 larvae were incubated with 250 µM of ascorbic acid or 0.1% ethanol as the solvent control for 40 h at 20°C. Subsequently, ascorbic acid-pretreated and control worms were divided into two aliquots and treated with or without 2 ppm of DEHP for 24 h at 20°C. (A) Relative sizes of fluorescent puncta for cell bodies of AFD sensory neurons. (B) Relative fluorescence intensities in cell bodies of AFD sensory neurons. Relative sizes of fluorescent puncta and relative fluorescence intensities were calculated by normalizing to that of the control. Approximately 30 worms from each treatment, at each time point, were randomly selected for analysis. The tests were performed a minimum of 3 times. The results were presented as the mean ± standard errors of mean (SEM). Differences between the populations were considered significant at *P*<0.05 by one-way ANOVA and the LSD post-hoc test. “Ctrl”, worms grown on a normal diet; “Ascorbic acid”, worms grown with ascorbic acid supplementation; “DEHP”, worms grown on a normal diet followed by DEHP exposure; “Ascorbic acid/DEHP”, worms with ascorbic acid pretreatment and followed by DEHP exposure.

We further explored the possible role of oxidative stress on DEHP-induced damage to AFD thermosensory neurons in *C. elegans*. Transgenic L1 larvae (P*gcy-8*::GFP) were pretreated with 250 µM ascorbic acid for 40 h at 20°C, followed by an DEHP exposure at the concentration of 2 ppm for 24 h. The results showed that ascorbic acid pretreatment significantly increased the number of DEHP reduced-fluorescence puncta, and relative GFP intensities of cell bodies in AFD sensory neurons (Figures 7A and 7B). The results showed that 2 ppm of DEHP exposure caused damage to the AFD thermosensory neurons in *C. elegans*, and that such damage can be prevented with a pretreatment of the antioxidant ascorbic acid.

## Discussion

Phthalate esters, widely used in flexible plastics and consumer products, are ubiquitous environmental contaminants, and numerous organisms are thus exposed to various levels of phthalates in their natural habitat. Studies of rodent models strongly implicate high-dose exposure to certain phthalates causing developmental and reproductive defects [12]. Therefore, in the *C. elegans* model, we initially used concentrations of DEHP (0.02, 0.2, 2, and 20 ppm), DBP (10, 100, 500, and 1000 ppm), and DIBP (1, 10, 100, and 1000 ppm) to induce these reproductive defects. The LOAELs for DEHP, DBP, and DIBP to cause significant brood size defects in *C. elegans* are 2, 500, and 100 ppm, respectively (data not shown). It has been suggested that the general potency of response on reproductive development in rodent models is DEHP > DBP [37]. Thus, the observed reproductive defects for DEHP and DBP in C. *elegans* are in agreement with those in rodent models. Based on the results of the reproductive toxicity test, DEHP (2 and 20 ppm), DBP (500 and 1000 ppm), and DIBP (100 and 1000 ppm) were used to examine the neurotoxic effects in *C. elegans*.

There is limited, but growing, evidence linking certain phthalates exposure to neurobehavioral outcomes. However, the effects and mechanisms of phthalates toxicity on neuronal functions require further explanation. Endpoints of body bending, head thrashing, and reversal frequency were used to evaluate the locomotor behavioral defects caused by phthalates exposure in *C. elegans*. The results showed that exposure to the phthalates (DEHP, DBP, and DIBP) at certain concentrations caused severe deficits in locomotor behaviors in *C. elegans* (Figure 1). A significant decrease in body bends, head thrashes, and reversal frequency were observed in wild-type *C. elegans*, exposed to all examined concentrations of phthalates, when compared with worms not treated with phthalates (Figures 1A, 1B, and 1C). Our data suggest that the endpoints of body bending, head thrashing, and reversal frequency are useful indices for the evaluation of phthalates-induced neurotoxicity in nematodes.

The thermotactic behaviors of *C. elegans* allow it to navigate spatial thermal gradients in an experience-dependent manner [38]. A number of neurons, including AFD, AWC, AIY, ASI have been identified to have roles in thermotaxis and the bilateral AFD neurons are a major thermosensory neuron type in *C. elegans* [28], [39]–[41]. Exposure to DEHP (20 ppm), DBP (500 and 1000 ppm), and DIBP (100 and 1000 ppm) caused severe neurotoxicity, affecting thermotactic behavior in nematodes, compared with that in the controls (Figure 2). This observation further supports the notion that exposure to high concentrations of phthalates may induce severe deficits in locomotor behaviors in nematodes, as shown in Figure 1. Moreover, this implies that phthalates exposure at high concentrations may result in multiple neurotoxic effects on the behaviors of exposed animals.

The *C. elegans* guanylyl cyclase genes *gcy-8, gcy-18*, and *gcy-23*, upstream of TAX-4, regulate thermotaxis through the AFD thermosensory neurons [30], [31]. *gcy-8* localizes to sensory endings and is expressed exclusively in the AFD thermosensory neurons [30]. Therefore, P*gcy-8*::GFP could be used as a specific fluorescent marker that labels the AFD sensory neurons [30]. A previous report used the altered size of fluorescent puncta, and relative fluorescence intensities of cell bodies in AFD neurons in P*gcy-8*::GFP to examine the effects of metal exposure on neuronal development [42]. In this study, phthalates exposure caused significant decreases in the size of fluorescent puncta, and in the GFP fluorescence intensity of cell bodies in AFD sensory neurons in *C. elegans* (Figures 3B and 3C).

Because the observed deficits by phthalates in the thermotaxis assays may be influenced by general locomotion behaviors of the examined nematodes, we investigated the effects of DEHP exposure at different concentrations (0, 0.2, 1, 2, 20 ppm) on locomotor behaviors and themotaxis assays. The results showed that treatments with 0.2 and 1 ppm of DEHP did not noticeably influence locomotor behaviors (body bends, head thrashes, and reversal frequencies) of nematodes (Figures S1A, S1B, and S1C). In contrast, treatments with 0.2 and 1 ppm of DEHP significantly inhibited isothermal tracking (IT) behavior to cultivation temperature (20°C) (Figure S1D). These data suggest that the observed deficits in thermosensation behavior in 0.2 and 1 ppm of DEHP treated nematodes were not due to the alterations of general locomotor behaviors of nematodes. This implies that general locomotor behavior is not disrupted by phthalates to the extent that general taxis behavior is disrupted. Moreover, this suggests that AFD defect is consistent with the observed thermotaxis results. However, other neurons might be involved and may also be affecting thermotactic behaviors altered by phthalates exposure.

It has been suggested that exposure to higher concentrations of metals (Hg, Cu, Ag, Cr, and Pb) results in a significant reduction in relative intensities and relative lengths of sensory endings in AFD neurons, and a significant reduction in the relative mRNA levels of TTX-1, TAX-2, TAX-4, and CEH-14, compared with controls [42], [43]. When nematodes were exposed to 2 ppm of DEHP, the mRNA level of TTX-1, a transcription factor that mediates expression of *gcy-8* [30], was decreased by approximately 40%, when compared with non-exposed controls (Figure 4), which would decrease the *gcy-8*::GFP level. Furthermore, mRNA levels of genes (TAX-2, TAX-4, and CEH-14) that are required for the differentiation and function of AFD neurons were significantly decreased when nematodes were exposed to 2 ppm of DEHP (Figure 4). Therefore, exposure of *C. elegans* to DEHP might cause a toxic effect on a molecular basis by influencing the expression of most of the genes that are required for the differentiation and function of AFD neurons (Figure 4).

We further explored a mechanism that might explain the manner in which the phthalates DEHP, DBP, and DIBP induced neurotoxicity on locomotor and thermotactic behaviors, and AFD neurons in *C. elegans*. Several studies have shown that DEHP produced free radicals and decreased GPx1 activity [44], [45]. The accumulation of oxidative damage to biomolecules has been implicated in the pathogenesis of a variety of neurondegenerative diseases [46]. Moreover, increased oxidative stress has been related to severely impaired learning behavior, and modestly reduced motor activity [34], [35]. Therefore, oxidative stress might be considered a crucial factor in phthalates-induced neurotoxicity. In this study, when the worms were exposed to all of the examined concentrations of DEHP, DBP, and DIBP, the intracellular ROS levels were significantly increased compared with those in the control (Figure 5A).

Our data further demonstrate that after antioxidant ascorbic acid pretreatment, the intracellular ROS production by DEHP exposure was decreased (Figure 5B). Moreover, pretreatment of ascorbic acid ameliorated the locomotor and thermotactic behavior defects, and protected the damage of AFD neurons by DEHP exposure (Figures 6 and 7). This implies that, once the oxidative stress is blocked or suppressed in DEHP-exposed nematodes, the decrease in locomotor behaviors and thermotactic reaction to cultivation temperature, and the structural defects of AFD sensory neurons caused by DEHP exposure in *C. elegans* can be effectively prevented. This suggests that phthalates exposure induced a significant increase of intracellular ROS production and thereby possibly disturbed the antioxidant defense systems in *C. elegans*, which in turn caused damage in neuronal systems and led to neurobehavioral abnormalities. Furthermore, DEHP, DBP, and DIBP induce a suite of neurobehavioral declines, suggesting that DEHP, DBP, and DIBP may disrupt the neuronal systems of *C. elegans* via a similar mode of action.

In conclusion, we showed that exposure to certain phthalates (DEHP, DBP, and DIBP) induced behavioral defects, including alterations to body bending, head thrashing, reversal frequency, and thermotaxis in *C. elegans*. Moreover, exposure to these phthalates caused changes in the morphological AFD sensory neurons, and the expression of most genes required for the differentiation and function of AFD neurons. Oxidative stress plays a critical role in the phthalates-induced neurotoxic effects observed in *C. elegans*.

## Materials and Methods

### Chemicals

All chemicals were obtained from Sigma-Aldrich Chemicals Co. (St. Louis, MO, USA) unless otherwise stated. DEHP, DBP, and DIBP were dissolved in 99% ethanol.

### Nematode strains and growth conditions

Nematodes used in this study were wild-type N2; DA1267 (*lin-15*(n765); dEx1267 [*lin-15*(+) *gcy-8*::GFP]) labeling the AFD neurons. All *C. elegans* strains and the *Escherichia coli* OP50 strain were obtained from the *Caenorhabditis* Genetics Center (CGC) (University of Minnesota, MN, USA), which is funded by the NIH National Center for Research Resources. The *C. elegans* strains were maintained and assayed (unless otherwise stated) at 20°C, on nematode growth medium (NGM) agar plates, seeded with a lawn of *E. coli* OP50 [47]. Synchronization of worm cultures was achieved using a bleaching buffer (0.45 M NaOH, 2% HOCl) treatment of gravid hermaphrodites [48].

### Locomotor behavior assays

For locomotor behavior assays, synchronized wild-type L1 larvae were incubated in liquid S-basal containing *E. coli* OP50 bacteria, at 10^9^ cells/mL in the absence or presence of 250 µM ascorbic acid, or 0.1% ethanol as the solvent control, for 40 h, at 20°C. Subsequently, L4-stage worms were incubated in K-medium, with or without phthalates, for 24 h at 20°C. The locomotor behavior assays were then performed. Various concentrations of DEHP (2 and 20 ppm), DBP (500 and 1000 ppm), DIBP (100 and 1000 ppm), or ethanol as the solvent control, were selected for locomotor behavior analysis. The behavior analysis is based on head thrash frequency, body bend frequency, and reversal frequency assay.

The body bend frequency assay was adapted from previous studies [26], [43]. The worms were washed with K-medium 3 times and subsequently transferred to a second plate and scored for the number of body bends in an interval of 20 s. A body bend was counted as a change in direction of the part of the worm corresponding to the posterior bulb of the pharynx along the Y-axis, with the assumption that the worm was traveling along the X-axis. Thirty nematodes were examined per treatment. The tests were performed a minimum of 3 times.

The head thrash frequency assay was adapted from previous studies [26], [43]. The worms were washed with K-medium 3 times. Each worm was transferred into 60 µL K-medium on the top of the agar. After a recovery period of 1 min, the head thrashes were counted for 1 min. A thrash was defined as a change in the direction of bending at the mid body. Thirty nematodes were examined per treatment. The tests were performed a minimum of 3 times.

The reversal frequency assay was adapted from previous studies [26], [43], [49], [50]. The control and treated worms were washed with K-medium 3 times. Worms were allowed to crawl away from any adherent food, at which point they were transferred to the uncoated NGM plates for reversal counting at 20°C. A period of 1 min elapsed prior to scoring, so that worms could recover from the transfer. Each worm was observed for 3 min, during which time any change from forward to backward movement, including an omega turn was scored as a reversal [51], [52]. A minimum of 30 nematodes was examined per treatment. The tests were performed a minimum of 3 times.

### Thermotactic assays

The thermotactic assay method was adapted from previous studies [53], [54]. Synchronized wild-type L1 larvae were incubated in liquid S-basal containing *E. coli* OP50 bacteria, at 10^9^ cells/mL in the absence or presence of 250 µM ascorbic acid, or 0.1% ethanol as the solvent control, for 40 h, at 20°C. Subsequently, L4-stage worms were incubated in K-medium, with or without phthalates, for 24 h at 20°C. Various concentrations of DEHP (2 and 20 ppm), DBP (500 and 1000 ppm), DIBP (100 and 1000 ppm), or ethanol as the solvent control, were selected for thermotactic analysis. The control and treated worms were washed with K-medium 3 times and placed onto uncoated NGM plates. Individual worms were then deposited onto a 9 cm TTX plate (2% agar, 0.3% NaCl, and 25 mM potassium phosphate buffer (pH 6.0)).

A radical temperature gradient was created by placing a vial containing frozen glacial acetic acid on the bottom of the TTX plate for 50 min at 25°C in the presence of a constant humidity of 60%. Upon removal of the nematodes from the plate, the tracks on the plate were photographed and analyzed. A trace is considered as isothermal if more than half of the trace length left on the agar surface by a single worm is circular, or presents an arc of a circle near the isotherm of the cultivation temperature. Each datum represents a minimum of 30 independent assays.

### Measurement of intracellular reactive oxygen species

Synchronized wild-type L1 larvae were incubated in liquid S-basal containing *E. coli* OP50 bacteria, at 10^9^ cells/mL in the absence or presence of 250 µM ascorbic acid, or 0.1% ethanol as the solvent control, for 40 h, at 20°C. L4-stage worms were subsequently incubated in K-medium with or without phthalates for 24 h at 20°C. Intracellular reactive oxygen species (ROS) in *C. elegans* were then measured using 2′,7′-dichlorodihydrofluoroscein diacetate (H_2_DCFDA). One hundred nematodes were broken up using sonication after each treatment, and the worm lysates were collected for the ROS measurement [55]. The worm samples were incubated with H_2_DCFDA (at a final concentration of 100 µM in phosphate buffered saline (PBS) in an FLx800 Microplate Fluorescent Reader (Bio-Tek Instruments, Winookski, VT, USA), for quantification of fluorescence with excitation at 485 nm and emission at 530 nm. The samples were read for 3 h. The tests were performed a minimum of 3 times.

### Analysis of fluorescence levels in transgenic strain DA1267

Synchronized DA1267 L1 larvae were incubated in liquid S-basal containing *E. coli* OP50 bacteria, at 10^9^ cells/mL in the absence or presence of 250 µM ascorbic acid, or 0.1% ethanol as the solvent control, for 40 h, at 20°C. Subsequently, L4-stage DA1267 worms were incubated in K-medium, with or without phthalates, for 24 h at 20°C. After treatment, the expression of *gcy-8* in each treatment group was directly measured by observing the fluorescence of the reporter green fluorescent protein (GFP).

The relative sizes of fluorescent puncta for cell bodies in AFD neurons in DA1267 were measured as the maximal radius for assayed fluorescent puncta. The relative fluorescence intensity of the cell bodies in AFD neurons of DA1267 worms was obtained by integrating pixel intensity. A minimum of 30 randomly selected worms from each set of experiments was mounted onto microscope slides coated with 3% agarose, anaesthetized with 2% sodium azide, and capped with coverslips. Epifluorescence images were captured using an epifluorescence microscope (Leica, Wetzlar, Germany) with a suitable filter set (excitation, 480±20 nm; emission, 510±20 nm) and a cooled charge coupled device (CCD) camera. The images were captured, and the GFP fluorescence of cell bodies in AFD neurons was quantified using Image-Pro Plus software (Media Cybernetics, Bethesda, MD, USA).

### Real-time quantitative reverse-transcription polymerase chain reaction analysis

Wild-type worms were treated and prepared as previously described. After DEHP treatment, total RNA from adult worms was isolated using TRIzol, according to the manufacturer's instructions (Invitrogen, Carlsbad, CA, USA), and cDNA was synthesized using Super-Script III, First-strand synthesis super-Mix for quantitative reverse-transcription polymerase chain reaction (qRT-PCR) (Invitrogen). The qRT-PCR was performed on a Step One real-time cycler (Applied Biosystems, Carlsbad, CA, USA) using a SYBR Green qPCR kit (Affymetrix, Inc., Cleveland, Ohio, USA). The qRT-PCR primers were designed for TTX-1 (forward: 5′-TCGGGAACGGACCACATTTA-3′; reverse: 5′-CTTCT GCTGCCTGGCCTTT-3′), TAX-2 (forward: 5′-ACATTTCATCCGTATGGTCGTTT-3′; reverse: 5′-CCGTGGTTTGATTAGCAGCAT-3′), TAX-4 (forward: 5′-TATCCGGATGCACG AAAGCT-3′; reverse: 5′-GCTTGAGTGCTCCACGATGA-3′), CEH-14 (forward: 5′-CCGGTGGAAGTCCTCAAATC-3′; reverse: 5′-GGTGTCTGCTCTCTGGAGTGAA-3′), and Y45F10D.4 (forward: 5′-GTCGCTTCAAATCAGTTCAGC-3; reverse: 5′-GTTCTTGTCAAGTGATCCGACA-3′). The relative quantities of mRNA were determined using comparative cycle threshold methods, and were normalized against the mRNA of Y45F10D.4 [56], which encodes a putative iron-sulfur cluster assembly. The fold change of the mRNA level was normalized to that observed in non-exposed control samples. The test was performed 3 times.

### Data analysis

Statistical analysis was performed using SPSS Statistics 17.0 Software (SPSS, Inc., Chicago, IL, 2008). The results are presented as the mean ± standard errors of mean (SEM). The statistical significance of differences between the populations was determined using one-way ANOVA and LSD post hoc test. Differences were considered significant at *P*<0.05 (see figures).

## Supporting Information

Figure S1 **Effects of DEHP exposure on locomotor behaviors and thermotaxis in** ***C. elegans*** **.** Synchronized wild-type L1 larvae were incubated in liquid S-basal containing *E. coli* OP50 bacteria, at 10^9^ cells/mL or 0.1% ethanol as the solvent control, for 40 h, at 20°C. Subsequently, L4-stage nematodes were incubated in K-medium, with and without DEHP (0, 0.2, 2, 2, and 20 ppm) for 24 h at 20°C. (A) The number of body bends in 20 s, (B) the number of head thrashes in 1 min, (C) the reversal frequency in 3 min, and (D). percentage of worms performing isothermal tracking (IT) behavior at the cultured temperature (20°C) Approximately 30 worms from each treatment, at each time point, were randomly selected for scoring. The tests were performed a minimum of 3 times. The results were presented as the mean ± standard errors of mean (SEM). Differences compared to the control (0 ppm, 0.1% ethanol) were considered significant at *P*<0.05 by one-way ANOVA and the LSD post-hoc test.
(TIFF)
