# Correction: Phthalates Induce Neurotoxicity Affecting Locomotor and Thermotactic Behaviors and AFD Neurons through Oxidative Stress in *Caenorhabditis elegans*

**DOI:** 10.1371/journal.pone.0099945

**Published:** 2014-06-04

**Authors:** I-Ling Tseng, Ying-Fei Yang, Chan-Wei Yu, Wen-Hsuan Li, Vivian Hsiu-Chuan Liao

The authors would like to acknowledge that the article contains overlap in text with that from our previous publication [1] (reference 43 in the original article) and fragments in the text that overlap word-for-word with text from our previous publication [2] and a publication in Environ Toxicol Pharmacol [3] (reference 42 in the original article).

The authors apologize for the overlap, for omitting to cite the publication [2] and for not properly quoting the text from [1, 2, 3]. The overlap in text relates to some of the information described in the Introduction section and to the description of the methodology under the Materials and Methods section.

However, it should be noted that the identified issues have no bearing on the results and conclusions of the study.
