# Supplementary figures and images for: Phthalates Induce Neurotoxicity Affecting Locomotor and Thermotactic Behaviors and AFD Neurons through Oxidative Stress in Caenorhabditis elegans
Source: PLoS One. 2013 Dec 12;8(12):e82657. doi: 10.1371/journal.pone.0082657 (PMC3861438; doi:10.1371/journal.pone.0082657)

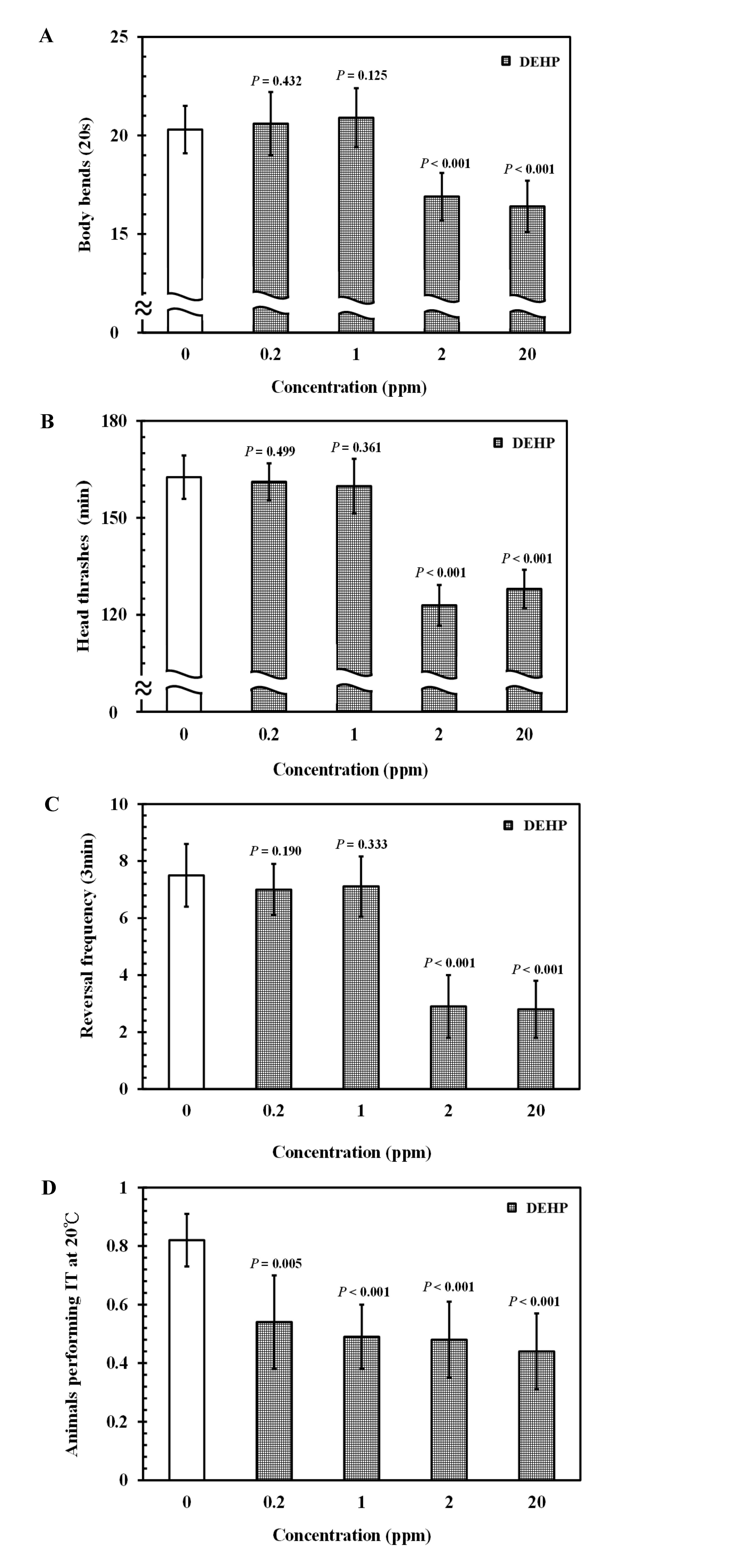

Supplement: Figure S1 — Effects of DEHP exposure on locomotor behaviors and thermotaxis in C. elegans . Synchronized wild-type L1 larvae were incubated in liquid S-basal containing E. coli OP50 bacteria, at 109 cells/mL or 0.1% ethanol as the solvent control, for 40 h, at 20°C. Subsequently, L4-stage nematodes were incubated in K-medium, with and without DEHP (0, 0.2, 2, 2, and 20 ppm) for 24 h at 20°C. (A) The number of body bends in 20 s, (B) the number of head thrashes in 1 min, (C) the reversal frequency in 3 min, and (D). percentage of worms performing isothermal tracking (IT) behavior at the cultured temperature (20°C) Approximately 30 worms from each treatment, at each time point, were randomly selected for scoring. The tests were performed a minimum of 3 times. The results were presented as the mean ± standard errors of mean (SEM). Differences compared to the control (0 ppm, 0.1% ethanol) were considered significant at P<0.05 by one-way ANOVA and the LSD post-hoc test. (TIFF) [file pone.0082657.s001.tiff]
